# Cardiovascular Diseases: From Pathophysiology to Novel Therapeutic Approaches

**DOI:** 10.3390/biomedicines14030503

**Published:** 2026-02-25

**Authors:** Affuso Flora, Serafino Fazio

**Affiliations:** 1 Independent Researcher, 80129 Naples, Italy; flora.affuso70@gmail.com; 2 Department of Internal Medicine, School of Medicine, Federico II University, Via Sergio Pansini 5, 80131 Naples, Italy

Despite advances in the prevention and treatment of cardiovascular diseases over the past three decades, they remain the leading cause of death in both developed and developing countries [1]. Furthermore, heart failure (HF), the common endpoint of most heart diseases, is the leading cause of hospitalization in individuals over 65, resulting in enormous healthcare costs [2]. The studies presented in this Special Issue provide important information in different fields of cardiovascular diseases. They range from vascular to cardiac and renal diseases, highlighting some peculiarities in determining pathophysiological mechanisms and therapeutic possibilities. For example, one study explores the long-term prognosis and impact factors in children with vasovagal syncope (VVS) receiving metoprolol therapy, showing that, after metoprolol treatment, 38.1% of children with VVS experienced syncope recurrence during a follow-up period of about 5 years. Moreover, a prognostic index derived using heart rate variability, standard deviation of all NN intervals, triangular index, and very low frequency was found to be associated with individual long-term prognosis [3]. Another manuscript based on a retrospective study and literature review shows the efficacy of revascularization interventions on complex lesions such as TASC C and D using endovascular procedures such as balloon angioplasty, stenting, and the use of drug-eluting stents [4]. Another review describes the multitude of underlying mechanisms of ischemic–reperfusion injury, also identifying current knowledge gaps and new therapeutic interventions [5]. Another interesting study published in this Special Issue highlights the sex-related differences of two commonly used antiplatelet drugs, prasugrel and ticagrelor, showing that females treated with prasugrel exhibited greater platelet aggregability induced by ADP stimulation than males; this did not occur with ticagrelor [6]. A further review article shows how a new technique, computed tomography-derived fractional flow reserve, that assesses both the anatomical and functional significance of coronary lesions is expected to become superior to other techniques in the individualized treatment of coronary artery disease [7]. Another interesting review explores the differences between two different pacing modes (Apex vs. Septum), concluding that, although the conduction system pacing approach represents more physiological pacing, right ventricular apex stimulation will continue to be a necessary and reliable option in current clinical practice [8]. A further review article analyzes the recent advances in nanoparticle-based therapies for counteracting atherosclerosis development, selectively targeting cellular players (such as macrophages, endothelial cells, and vascular smooth muscle cells) in plaque pathogenesis [9]. Another manuscript published in this Special Issue compares the effects of old vs. new diuretics on the kidney, and also demonstrates the efficacy of combination therapies [10]. An experimental study, performed using rat precision-cut lung slices, examined the effects of vericiguat and riociguat on pulmonary arteries, veins, and airways, showing that these drugs, in this context, dilated the pulmonary arteries, induced bronchodilatation, and reduced inflammation [11].

A further interesting article shows the results of an experimental study assessing which circulating macrovesicles (MVs) were present in patients with acute coronary syndrome vs. healthy controls. The researchers conclude that reduced levels of miR-126-5p and miR-223-3p in circulating MVs are strongly associated with impaired coronary flow, and that these miRNAs should be considered potential biomarkers for ACS risk stratification and therapeutic targeting [12]. The last article is a case report description of a patient with a renal abscess associated with SGLT2 inhibitor administration in a person without other previous predisposing risk factors, underlining that, although rare, this important complication could occur during therapy with SGLT2 inhibitors [13].

The collection of articles in this Special Issue describes important new pathophysiological mechanisms underlying the development of cardiovascular diseases and indicates new diagnostic and therapeutic possibilities.
